# New Benzotrithiophene-Based Molecules as Organic P-Type Semiconductor for Small-Molecule Organic Solar Cells

**DOI:** 10.3390/ma16103759

**Published:** 2023-05-16

**Authors:** Cristian Castillo, Andrés Aracena, Luis Ballesteros, Gloria Neculqueo, Loik Gence, Franck Quero

**Affiliations:** 1 Laboratorio de Nanocelulosa y Biomateriales, Departamento de Ingeniería Química, Biotecnología y Materiales, Facultad de Ciencias Físicas y Matemáticas, Universidad de Chile, Avenida Beauchef 851, Santiago 8370456, Chile; 2 Instituto de Ciencias Naturales, Facultad de Medicina Veterinaria y Agronomía, Universidad de Las Américas, Sede Santiago, Campus La Florida, Avenida Walker Martínez 1360, La Florida, Santiago 8240000, Chile; 3 Instituto de Ciencias Químicas Aplicadas, Grupo de Investigación en Energía y Procesos Sustentables, Universidad Autónoma de Chile, Av. El Llano Subercaseaux 2801, San Miguel, Santiago 8910060, Chile; 4 Centro de Materiales para la Transición y Sostenibilidad Energética, Comisión Chilena de Energía Nuclear, Santiago 7600713, Chile; 5 Functional Materials & Devices Lab, Pontificia Universidad Católica de Chile, Santiago 8940000, Chile; 6 Instituto de Física, Pontificia Universidad Católica de Chile, Avenida Vicuña Mackenna 4860, Santiago 8940000, Chile

**Keywords:** benzotrithiophene derivative, organic semiconductors, π-stacked assemblies, small molecule, organic solar cells

## Abstract

A new benzotrithiophene-based small molecule, namely 2,5,8-Tris[5-(2,2-dicyanovinyl)-2-thienyl]-benzo[1,2-b:3,4-b′:6,5-b″]-trithiophene (DCVT-BTT), was successfully synthesized and subsequently characterized. This compound was found to present an intense absorption band at a wavelength position of ∼544 nm and displayed potentially relevant optoelectronic properties for photovoltaic devices. Theoretical studies demonstrated an interesting behavior of charge transport as electron donor (hole-transporting) active material for heterojunction cells. A preliminary study of small-molecule organic solar cells based on DCVT-BTT (as the P-type organic semiconductor) and phenyl-C61-butyric acid methyl ester (as the N-type organic semiconductor) exhibited a power conversion efficiency of 2.04% at a donor: acceptor weight ratio of 1:1.

## 1. Introduction

There is a growing need for more environmentally friendly and renewable energy since its access still remains challenging. Among natural energy sources, the limitless potential of sunlight is still a highly relevant strategy for fully realizing its potential. In this context, organic solar cells (OSC) are currently attracting much attention due to their wide range of optoelectronic properties, lower production cost, ease of processing, and a large variety of applications for the design of flexible devices [1,2,3,4,5] with the challenge of increasing the power conversion efficiency (PCE) [6,7]. Even nowadays, the access to clean, sustainable, and affordable energy [8,9,10,11,12,13,14] remains the seventh goal of the world agenda 2030 for sustainable development.

In contrast to the widely studied polymer-based OSC, small-molecule organic solar cells (SM-OSC) have demonstrated several outstanding advantages, including their well-defined structure and precise molecular weight, ease of purification, and as a result, little batch-to-batch variation [15,16]. Recently, high power conversion efficiency [17] has been achieved for SM-OSC, indicating that their PCE could reach values similar to polymer solar cells [2]. These PCE values can be obtained by utilizing the combination of adequate molecule design and device optimization. The PCE of photovoltaic systems is determined by three parameters, namely open-circuit voltage (V_OC_), short-circuit current density (J_SC_), and fill factor (FF). Generally, SM-OSC possess a high V_OC_ and FF over 50% [18,19,20] and improving the J_SC_ without sacrificing V_OC_ and FF is one of the effective strategies to attain a high PCE for OSCs. There are many methods to improve the J_SC_, such as active layer morphology, which can be controlled by adding additives and modification of the buffer layer [21]. The direct and fundamental strategy to improve the J_SC_ is to design new electron donor (D) molecules with a broad light absorption range and high light harvesting efficiency. These properties allow the sunlight to be captured more efficiently, thus increasing the formation of charge carriers that are responsible for producing the photovoltaic effect.

**Scheme 1:**
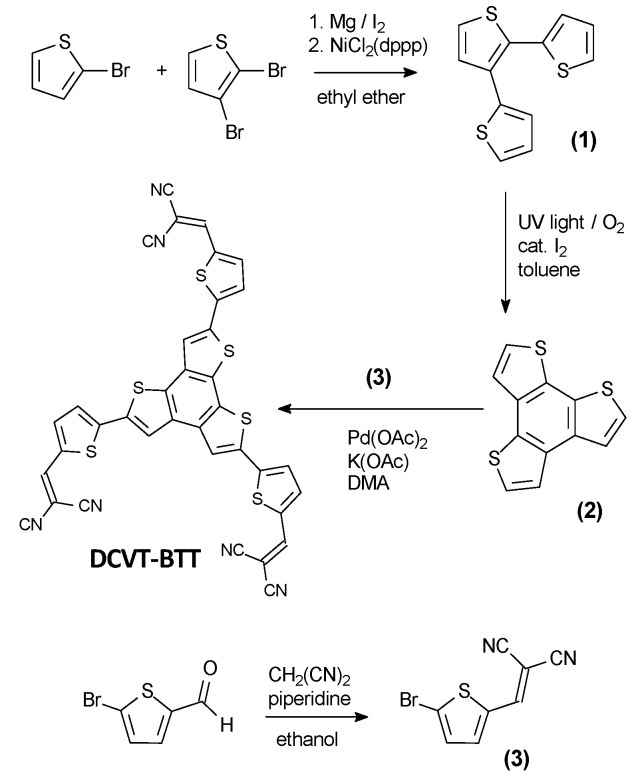
Synthetic routes for the preparation of DCVT-BTT compounds.

Another simplified approach for the design of new small molecules is by increasing conjugation length through the π-spacer. A suitable compound to be used as a spacer is the asymmetric core benzo [1,2-b:3,4-b′:6,5-b″]trithiophene (BTT), which has been used in several investigations as a molecular basis for the manufacture of new compounds with electron donor behavior [22,23,24,25]. Unlike the symmetric BTT ring, the cis arrangement of the sulfur atoms in asymmetric BTT facing each other in the π-spacer core (Scheme 1) may be further benefited in the intermolecular π-interaction, thus allowing enhanced hole extraction [22,23,24,26,27,28].

Another strategy used to modify the electronic properties of a molecule is the incorporation of specific functional groups with high electron-withdrawing potential, called acceptor groups (AG). These groups decrease the electronic bandgap, which corresponds to the difference in energy between the highest occupied molecular orbital (HOMO) and lowest unoccupied molecular orbital (LUMO), facilitating the exciton formation [29,30]. This originates from an electron-attracting effect that induces an increase in molecular polarizability, causing a push–pull effect [31,32]. One of the most used acceptor groups is dicyanovinylene (DCV), which in addition to fulfilling its structural function, offers a simple synthetic methodology. DCV has been used in various studies related to the synthesis and evaluation of small molecules, oligomers, polymers, and organic electronic systems [33,34]. In order to extend the light absorption range and induce an efficient solid-state packing and by considering the previously mentioned factors, we designed a small molecule that has an asymmetrical benzotrithiophene (BTT) unit as the π-spacer central core and three dicyanovinyl-thiophene (DCVT) acceptor group (Scheme 1). This was obtained using a new organic synthetic process referred to as direct (hetero)arylation (DHA) [35]. As expected, the final compound exhibited broad and solar redshift absorption in contrast to the core structure alone and can be used as electron donor active material in OSC devices with bulk heterojunction architecture using phenyl-C61-butyric acid methyl ester (PC_61_BM) as an electron acceptor active material.

## 2. Materials and Methods

All the chemical substances used in the present study were purchased from Sigma-Aldrich and Merck Chemicals, and were subsequently used without further purification. The solvents were distilled and dried by standard methods before use.

### 2.1. Chemical Synthesis

#### 2.1.1. Synthesis of 2,2′:3′,2″-Terthiophene **(1)**

A solution of 2-bromothiophene (3.6 g, 22 mmol) in 15 mL of ether was added dropwise over Mg turnings in 45 mL of ether under a controlled N_2_ atmosphere. The resulting mixture was subsequently refluxed for 12 h. The Grignard reagent was subsequently added dropwise into a mixture of 2,3-dibromothiophene (6.05 g, 0.025 mol) and Ni(dppp)Cl_2_ (65 mg, 0.12 mmol) in 25 mL of ether. The resulting brown solution was refluxed for 15 h and cooled down to room temperature. Finally, 150 mL of HCl 1M was added to stop the reaction. The ether layer was separated, washed with saturated NaCl, dried over MgSO_4_, and concentrated, resulting in a yellowish-brown oil. The oil was purified by flash chromatography silica gel, where hexane was used as the solvent. This resulted in obtaining 4.05 g of light cyan crystals. Mp: 39–40 °C. UV EtOH: λ_max_/nm: 303 (log ε = 4.04).

#### 2.1.2. Synthesis of Benzo[1,2-b:3,4-b′:6,5-b″]trithiophene (BTT) **(2)**

A mixture of 156.8 mg (0.6314 mmol) of **(1)** and 8.1 mg (0.0319 mmol) of iodine was dissolved in 40 mL of toluene. The mixture was stirred in a quartz-glass tube under gentle oxygen flow and 400 W mercury lamp illumination overnight. The mixture was washed with an Na_2_S_2_O_3_ solution (10 g in 50 mL of deionized water). The organic phase was dried over MgSO_4_ for 3 h and separated by chromatography column, where hexane was used as eluent. A white crystalline solid was obtained (125.3 mg, 0.516 mmol, yield = 81.0%). Mp: 157–158 °C. ^1^H-NMR (CDCl_3_): δ 7.50 (d, 1 H, J = 5.4 Hz), 7.51 (d, 1H, J = 5.1 Hz), 7.53 (d, 1H, J = 5.4 Hz), 7.63 (d, 1 H, J = 5.4 Hz), 7.76 (dd, 2H, J = 5.4 Hz, J = 1.8 Hz). ^13^C-NMR (CDCl_3_): δ 122.72, 122.89, 123.04, 124.61, 124.65, 125.22, 130.91, 131.22, 131.75, 132.42, 132.90, 132.95. UV–Vis (EtOH) λ_max_/nm: 288 (log ε = 4.21), 267 (log ε = 4.84).

#### 2.1.3. Synthesis of 5-Bromo-2-dicyanovinylthiophene **(3)**

Solutions of malononitrile (1.2 g, 18 mmol) and 5-bromothiophene-carbaldehyde (15 mmol) in ethanol (50 mL) were added in piperidine (1 drop). The solution was heated and refluxed for 1 h, subsequently cooled down to ambient temperature, and finally the solvent was removed under reduced pressure to obtain a crude dicyanovinyl compound. The resulting solids were recrystallized, resulting in a pale orange solid (91.2%). Mp: 157–158 °C. (eluent ethyl acetate/n-hexane). UV (CH_3_Cl): λ_max_ nm: 317.5 (log ε = 4.23). FT-IR (cm^–1^) 3310, 2224. ^1^H NMR (CDCl3): δ 7.25 (d, 1H, J = 4.0 Hz, 4-H), 7.51 (d, 1H, J = 4.0 Hz, 3-H), 7.75 (s, 1H, CH=C(CN)_2_).

#### 2.1.4. Synthesis of 2,5,8-Tris[5-(2,2-dicyanovinyl)-2-thienyl]-benzo[1,2-b:3,4-b′:6,5-b″]trithiophene (DCVT-BTT)

First, a mixture of **(2)** (500 mg, 1.7 mmol), potassium acetate (334 mg, 3.4 mmol) and palladium acetate (1.9 mg, 8.4 mmol) was prepared, to which compound **(3)** (419 mg, 3.74 mmol) and N,N-dimethylacetamide (6 mL) were subsequently added. This was performed, under a controlled N2 atmosphere, using a 100 mL capacity three-necked round-bottomed flask fitted with a thermometer, a magnetic stirrer, a condenser, and a rubber seal. The mixture was heated to 150 °C under vigorous stirring for 24 h, resulting in a dark red solution. The latter was cooled down to room temperature, filtered, washed with water, ethyl acetate as well as petroleum ether, and finally dried. A total of 430 mg of solid dark purple product was obtained corresponding to a yield of 71%. The chemical characterization by FT-IR (cm^−1^) and 1H NMR (CDCl3) is included in Supporting Information: Figure S9 and Figure S10, respectively.

### 2.2. DCVT-BTT Compound Characterization

Fourier-transform infrared spectra were obtained using a spectral resolution of 2 cm^−1^. A Perkin Elmer Model Spectrum One FT-IR spectrometer was utilized to obtain the spectrum. UV–visible spectra of DCVT-BTT were monitored using a Hewlett-Packard 8452-A diode array spectrophotometer. Measurements were performed in the liquid state, using acetone as solvent. ^1^H NMR spectra (400 MHz) were determined using a Bruker Model Advance Digital. The electrochemical properties of the DCVT-BTT compound were measured by cyclic voltammetry (CV) using a CHI-660E electrochemical workstation (Chenhua Instruments Co., Shanghai, China). All electrochemical experiments were performed using a conventional three-electrode system fitted with a glassy carbon electrode GCE (φ = 2 mm) as the working electrode, a platinum wire as the counter electrode, and an Ag/AgCl (sat. KCl) as the reference electrode. The solution utilized is a 0.1 M Bu_4_NPF_6_/CH_3_CN. The scan rate of 100 mV s^−1^ was used to perform the experiments.

### 2.3. Computational Methods

The BTT-TDCV structures (monomers and dimer) were calculated with an M06-2X functional and 6-31G (d,p) basis set. In addition, the BTT-TDCV monomer geometry optimization was performed with B3LYP/6-31G (d,p) for comparison. All these calculations were carried out using Gaussian 09 [36]. The electronic transitions in acetone as continuum were calculated by TD-DFT using the PCM method. The electron excitation properties were obtained with Multiwfn 3.8 software [37].

### 2.4. Organic Solar Cells Fabrication

**Figure 1:**
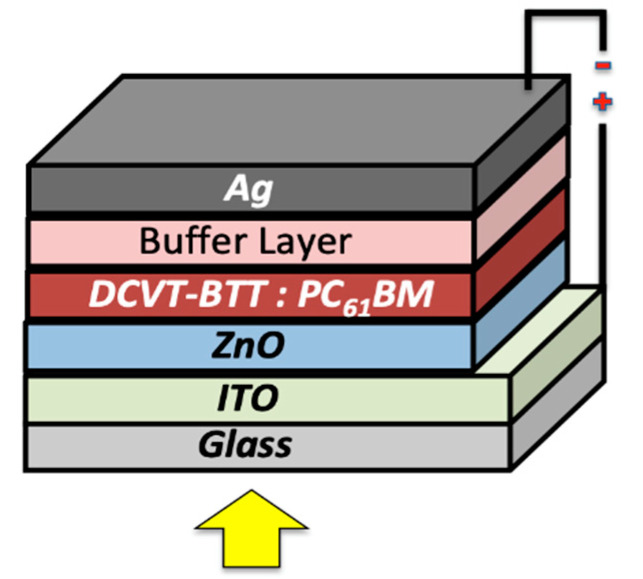
Schematic representation of the SM-OSC configuration used in the present study. The active layer corresponds to a bulk heterojunction system of donor and acceptor molecules.

All materials were deposited onto indium tin oxide substrates (ITO). The ZnO layer was prepared from a 50 g L^−1^ zinc acetate solution (Zn(OAc)_2_ × 2H_2_O) dissolved in 96% 2-methoxy ethanol and 4% ethanolamine and deposited as reported before in the literature [1,2]. 1,2-dichlorobenzene was used to dissolve both DCVT-BTT and PC_61_BM, separately, and each solution was stirred at 50 °C overnight. The solutions were subsequently combined, stirred at 70 °C for 2 h, and finally sonicated (at 50 °C for 30 min) prior to depositing the mixture by spin coating. Weight ratios of 1:2, 1:1 and 2:1 were obtained and prepared from the mixed DCVT-BTT:PC_61_BM solution having a concentration of 32 g L^−1^. Each organic active blend was spin coated, individually, at 600 rpm for 5 min using a WS-400B-6NPP/LITE spin coater (Laurell Technologies Corporation, Lansdale, PA, USA) under N_2_ flow. The spin-coated films were subsequently annealed at 110 °C for 10 min under vacuum. Both buffer layers and Ag anodes were fabricated using a vacuum evaporator (Edwards Auto 306, Edwards Vacuum LLC, New York, NY, USA) from a ceramic jar and an Mo boat, respectively. The successive depositions were carried out under vacuum (~6.10^−6^ mbar), while maintaining the growth rate constant (0.01 nm s^−1^). The buffer layer thickness was kept constant (~5 nm) for all cells. An Ag layer thickness of ~150 nm was formed by evaporation (shadow mask) to fabricate the OSC devices. Finally, for each cell, a layered structured of ITO/ZnO (25 nm)/DCVT-BTT:PC_61_BM (110 nm)/MoO_3_ (5 nm)/Ag (150 nm) was obtained [38] as shown in Figure 1 and as reported before in the literature [39,40,41].

### 2.5. Photovoltaic Characterization of Solar Cells

The current–voltage (I–V) characteristics in dark and under simulated AM 1.5 sunlight illumination (50 mW cm^−2^) were measured using a source meter (Keithley 2400, Artisan Technology Group Ltd., Champaign, IL, USA). The AM 1.5 sunlight illumination was produced with a filtered Xe-lamp in the Simulator Solar (Sciencetech SS 150W Ltd., London, ON, Canada). All measurements were carried out in the air and at room temperature, without encapsulation of the devices. Unfortunately, it was not possible to perform certified efficiency measurements in the frame of the present study. It must be highlighted, however, that the scope of the present work was not to obtain absolute efficiency values. Instead, an estimation of the relative efficiency resulting from the use of new buffer layers based on the newly synthesized benzotrithiophene-based small molecule is provided.

## 3. Results and Discussions

### 3.1. Molecular Design and Synthesis of Compounds

The synthetic route for the preparation of DCVT-BTT is reported in Scheme 1. The first step is the synthesis of terthiophene **(1)**, which involves a nickel-catalyzed Kumada coupling process occurring between Grignard reagent and 2-bromothiophene. The asymmetrical central core of BTT **(2)** was synthesized by oxidative photocyclization by irradiation with a mercury lamp 400 W of a diluted toluene solution of **(1)** under aerobic conditions in the presence of a catalytic amount of iodine. Finally, a carbon–carbon coupling of 5-bromo-2-dicyanovinylthiophene (Compound **(3)**) with **(2)** is produced by DHA with yields being typically higher than 80% [35,42,43].

### 3.2. Experimental UV–Visible Spectrum and Theoretical UV–Visible Electronic Transitions for DCVT-BTT

**Figure 2:**
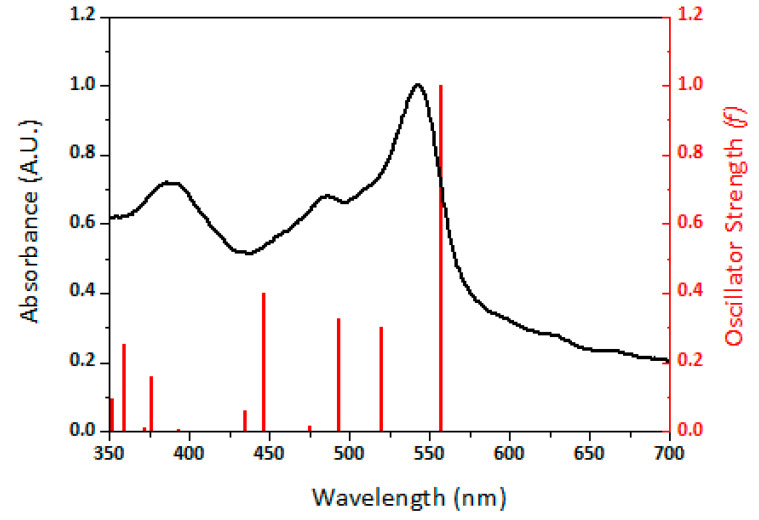
Comparison of a UV–visible spectrum of DCVT-BTT (black) with calculated oscillator strength values for DCVT-BTT, both obtained using acetone as solvent. The oscillator strength values were scaled to match the experimental spectrum.

Figure 2 reports a typical experimental UV–visible spectrum for the DCVT-BTT compound. One can observe a principal electronic transition at a wavenumber position of ∼557 nm, which corresponds to the HOMO-LUMO transition. In addition, Table 1 reports calculated values for oscillator strength with a maximum value being located at a wavelength of ∼543 nm corresponding to the lower energy band in the UV–Vis spectrum of DCVT-BTT. The wavelength position of ∼557 nm obtained experimentally is relatively close to the value of ∼543 nm obtained theoretically. One can also observe that the oscillator strength of the lower energy electronic transition (λ ∼543 nm) possesses higher relative intensity (more allowed) than the other electronic transitions. This is also observed experimentally in the UV–visible spectrum, where the major transition also has a higher relative intensity than the other electronic transitions with lower relative intensities.

**Figure 3:**
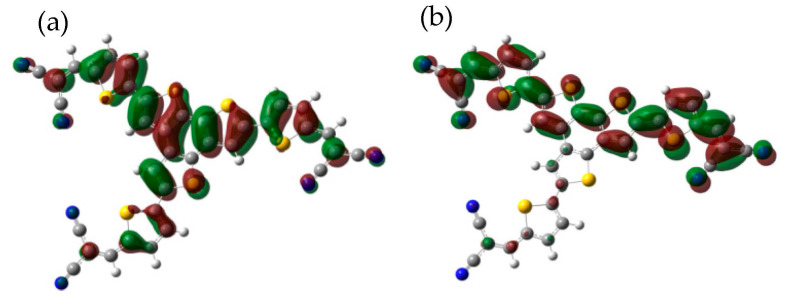
Frontier molecular orbitals of DCVT-BTT. (**a**) HOMO and (**b**) LUMO.

Figure 3a,b report the HOMO and LUMO frontier molecular orbitals of DCVT-BTT, respectively. One can see that the HOMO is centered on the BTT core and the outer thiophene rings. When this molecule absorbs a photon, one electron is ejected from an arm of the molecule from the HOMO to the LUMO, through the alternating double bonds and the electron-withdrawing nitriles. The theoretical energies of the HOMO and the LUMO, using the M06-2X functional, were found to be −7.12 eV and −3.05 eV in the gas phase, respectively. When using the B3LYP functional, however, energy values of −6.04 eV and −3.61 eV were obtained.

Oxidation and reduction potentials for the DCVT-BTT were quantified by cyclic voltammetry in solution state using a previously reported method [44] and the following equations:

HOMO = Ionization Potential = −(EOX + 4.4) eV(1)

LUMO = Electron Affinity = −(ERED + 4.4) eV(2)

An ITO electrode was used as the anode, with a potential value of −4.80 eV. An Ag electrode with a potential value of −4.74 eV was used as the cathode. A difference of 2.32 eV between the HOMO (calculated with the M062X functional) of DCVT-BTT and the ITO anode was found. When using the B3LYP functional, this difference was found to be 1.24 eV. In order to allow for ohmic contact, however, these energy differences should not exceed 0.3 eV for DCVT-BTT in solution. In a previous study of a BTT derivative RO-TriT, the calculated HOMO energy from a B3LYP to M06-2X level of theory was also found to produce differences [45]. The experimental difference between the HOMO of CV and the ITO anode was 0.26 eV, which favored the ohmic contact, resulting in photovoltaic behavior. This suggests that the injection of holes from DCVT-BTT to the ITO electrode and the injection of electrons from the LUMO level of DCVT-BTT to the LUMO level of PC_61_BM occurs.

**Figure 4:**
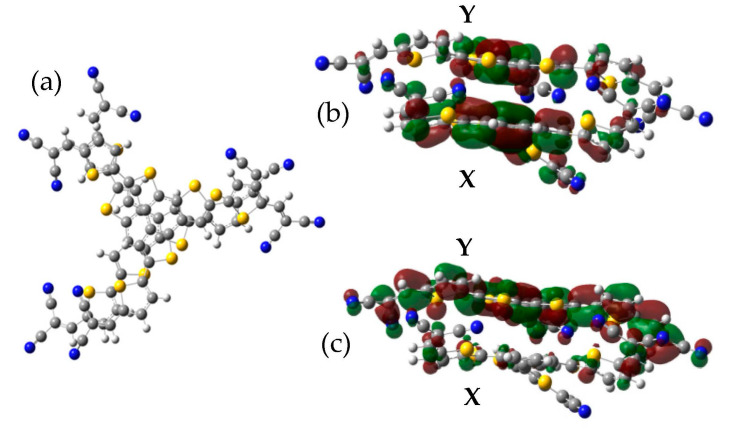
(**a**) Optimized molecular structure of DCVT-BTT dimer. Frontier molecular orbitals of DCVT-BTT dimer: (**b**) HOMO and (**c**) LUMO.

Discotic stacking in BTT derivatives has been previously reported in the literature [24,25,26,46,47,48,49]. Consequently, in the present study, the electron excitation and charge transport properties of DCVT-BTT as the monomer as a π-stacked dimer were calculated since the stacking interaction properties of DCV-BTT provide worthy information compared to the single molecule. Figure 4a shows the optimized π-stacked dimer of DCVT-BTT. The calculated interaction energy between monomers without the BSSE correction was found to be −8.28 Kcal/mol, suggesting great structural stability.

For the HOMO (Figure 4b) and the LUMO (Figure 4c) of DCVT-BTT, it is possible to observe that the HOMO is mainly centered on the bottom monomer (X) and partly on the top monomer (Y). Nevertheless, the LUMO is centered almost entirely on the top monomer (Y). This suggests that the monodirectional migration of one electron when the electronic HOMO-LUMO transition occurs from the bottom monomer (X) in HOMO to the LUMO in the top monomer (Y) of the dimer.

### 3.3. Electronic Excitation Properties of DCVT-BTT

Recently, electronic excitation properties have been studied for diverse organic molecules [50,51,52,53,54,55]. Table 2 displays the electron excitation properties such as Δr index [56], which measures the CT length during electron excitation. The D index is defined as the distance between the centroid of the hole and the electron along their corresponding directions and it characterizes the total CT length. The *t* index [57] measures the separation degree in relation to the hole and the electron distribution. The S index is useful to characterize the overlapping extent of holes and electrons. Exciton binding energy [58,59] is the hole–electron Coulomb attractive energy, which is closely related to the electron excitation characteristics. Among all, the most influential factor should be the D index. It is easy to understand that the larger the D index is, the greater the distance between the main distribution regions of the hole and the electron, and thus the weaker the Coulomb attractive energy. H index [57] is an overall measure, which reflects the breadth of the average distribution of the hole and the electron. The Λ index essentially measures the overlapping degree of the hole and the electron of the electron excitations [60]. The hole delocalization index (HDI) and the electron delocalization index (EDI) are pretty useful in quantifying the breadth of spatial distribution (i.e., degree of delocalization) of the hole and the electron, respectively.

For the dimer, the Δr index for the S0→S1 transition was to be 2.134 Å, which is higher than 2 Å and consequently can be considered as a charge transfer transition. For the monomer, however, this value is slightly below 2 Å (Table 2). A negative value for the t index implies no special separation between hole and electron but the value in the dimer is closer to zero than the value obtained for the monomer. D index for the dimer shows a greater value for the distance between centroid hole and electron than the monomer, in agreement with a smaller value for the exciton dissociation energy obtained for the dimer compared to the monomer. The S index values suggest that there is a major overlap of hole and electron for the monomer compared to the dimer. The H index is similar for both, indicating a large distribution of hole and electron for the S0→S1 transition. A large Λ value implies a high degree of overlap between hole and electrons, which is, however, not the case for both monomer and dimer. HDI and EDI are both larger for the monomer than the dimer. Finally, interfragmental charge transfer analysis for the S0→S1 excitation for the monomer and dimer resulted in large values in charge transfer percent (over 50%) with regard to the local excitation, suggesting a CT character for the S0→S1 transition. Another graphical representation of the electronic excitation for the DCVT-BTT monomer and dimer are provided in ESI (Figures S1–S8). All these features show a good charge transfer behavior when DCVT-BTT interacts with a light photon, typical for the behavior of an electron donor material in an organic solar cell.

### 3.4. Calculation of Charge Transport Properties for DCVT-BTT

Charge transport in discotic compounds mostly occurs by hopping mechanism [61,62]. This process is described as a self-exchange hole/electron transfer reaction between two neighboring molecules. The semiclassical Marcus theory defines the charge transfer rate constant kET at room temperature as

(3) $$k_{ET}=\frac{4\pi^{2}}{h}\;\frac{1}{\sqrt{4\pi \lambda k_{B}T}}\;t^{2}\;exp\;\left[\frac{-\lambda }{4k_{B}T}\right]\;$$

where *t* is the electronic coupling and *λ* the reorganization energy. The electronic coupling *t* (or charge transfer integral) is an extent of the strength of the electronic interactions between two contiguous molecules and it is dependent on the distance between molecules.

Here, we used the Koopman methodology to study the electronic coupling [63,64,65]. The reorganization energy *λ* is produced by fast changes in molecular geometry (the inner contribution) and by speed-reduced changes in polarization around the medium (the outer contribution). The latter contribution can be neglected [66] because these molecules represent a static organic crystal packing and the outer contribution is minimum. The inner reorganization energy *λ_i_* can be obtained by

(4) $$\lambda_{i}^{\left(A1\;hole\right)}=E^{\left(A1\right)}\left(M^{+}\right)-E^{\left(A1\right)}\left(M\right)$$

(5) $$\lambda_{i}^{\left(D2\;hole\right)}=E^{\left(D2\right)}\left(M\right)-E^{\left(D2\right)}\left(M^{+}\right)$$

(6) $$\lambda_{i}^{\left(A1\;electron\right)}=E^{\left(A1\right)}\left(M^{-}\right)-E^{\left(A1\right)}\left(M\right)$$

(7) $$\lambda_{i}^{\left(D2\;electron\right)}=E^{\left(D2\right)}\left(M\right)-E^{\left(D2\right)}\left(M^{-}\right)$$

(8) $$\lambda_{i}=\lambda_{i}^{\left(A1\;hole/electron\right)}+\lambda_{i}^{\left(D2\;hole/electron\right)}$$

The charge mobility (*μ*) shows how quickly a hole or electron can move through a semiconductor. It can be calculated by the Einstein equation [67,68]

(9) $$\mu =\frac{eD}{k_{B}T}=\frac{el^{2}}{k_{B}T}\;k_{ET}$$

For a one-dimensional system, it is possible to obtain *D*, where *l* is the distance between molecules and *k_ET_* is the charge transfer rate constant between contiguous molecules. Since the outer *λ_out_* can be neglected to obtain *k_ET_*, the charge mobilities should be considered as relative.

Table 3 shows that the *t* value for HOMO is larger than the *t* value for LUMO that is characteristic of face to face conformation of studied BTT derivatives [45]. *λ* hole and *λ* electron (both inners) satisfy the condition *λ* >> *t* [69,70], which is necessary for the hopping mechanism. *λ*_e_ > *λ*_h_ shows the typical tendency that promotes a greater mobility for holes than electrons. The calculated relative charge mobilities, in general, produce low values for holes and electrons, but the strong P donor character vs. N acceptor is clear in this organic semiconductor.

### 3.5. SM-OSCs Performance and Evaluation

**Figure 5:**
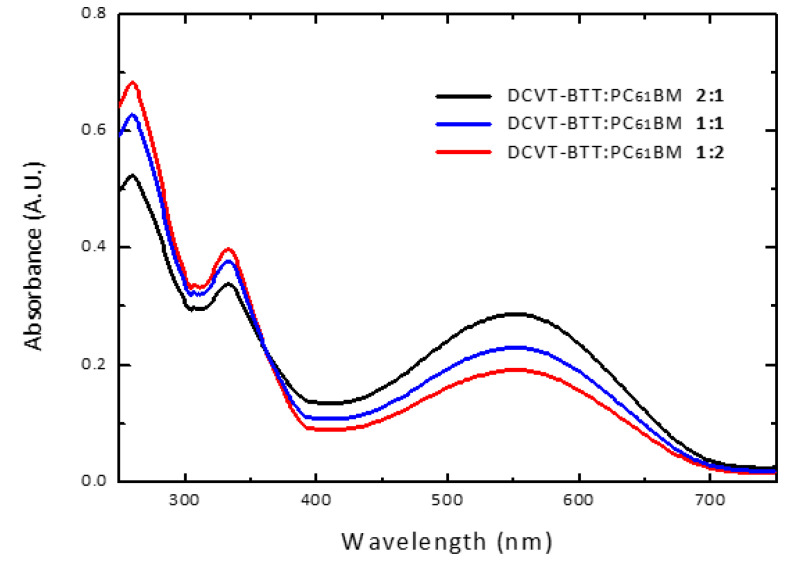
UV–visible absorption spectra of DCVT-BTT:PC_61_BM films onto quartz prepared at various D:A weight ratios (2:1, 1:1, and 1:2).

Solar cells were fabricated using DCVT-BTT (small molecule) as an electron donor, and PC_61_BM was used as the electron acceptor, which were deposited by spin coating using dichlorobenzene as the solvent. The SM-OSC devices were tested separately at room temperature in darkness and exposure to simulated AM 1.5G illumination at 100 mW cm^−2^. Different weight ratios of donor and acceptor molecules were used (2:1, 1:1, 1:2), and the photovoltaic effect was quantified for all compositions. In order to manufacture and measure the photovoltaic performances of DCVT-BTT, SM-OSC devices were fabricated in an inverted bulk heterojunction configuration of ITO/ZnO/DCVT-BTT:PC_61_BM/MoO_3_/Ag, based on the existing bibliography [38,39]. The UV–visible absorption spectra of DCVT-BTT:PC_61_BM active thin layers, prepared at different weight ratios, are reported in Figure 5. The absorption band of DCVT-BTT in solid state exhibited a broad peak that allows covering a wide area of the visible spectrum (from λ = 420 nm up to λ = 670 nm), permitting a much higher energy uptake due to the broader absorption wavelength range measured for all DCVT-BTT:PC_61_BM film compositions. Compared to the UV–visible spectra obtained for the small-donor molecule in acetone solution, the maximum wavelength in the visible spectrum of the films as well as its redshift (12 nm) also suggested an intermolecular interaction in the solid state. An increase in the maximum wavelength absorption for the DCVT-BTT:PC_61_BM films manufactured using a weight ratio of 2:1 was observed in the UV region, while for the fabricated films with a higher proportion of DCVT-BTT (1:1 and 1:2), a relative intensity increase in the absorption peak was observed in the visible region. Both results suggest than intensification of the optoelectronic properties is not occurring.

**Figure 6:**
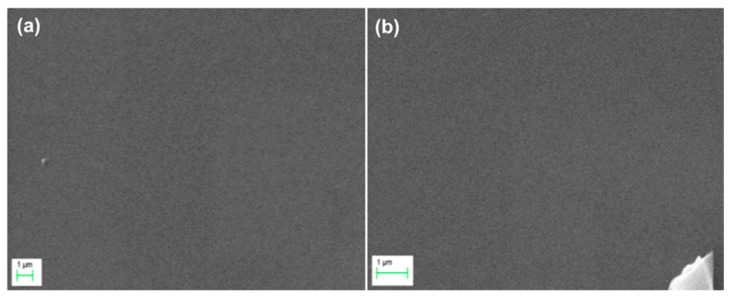
Scanning electron micrographs obtained from the surface of DCVT-BTT:PC_61_BM layer of OSC devices with donor (D) and acceptor (A) weight ratios of (**a**) D:A = 1:1 and (**b**) D:A = 2:1.

The structure and surface morphology of the active layer is essential for the adequate manufacturing of OSC systems. A high film quality allows a greater interconnection between the donors and acceptors species, thus allowing better interfacial contact with the buffer layer and electrode, which has been shown to improve photovoltaic properties [38,39,40,41]. SEM micrographs of the active donor: acceptor layer of OSC devices are reported in Figure 6. It is possible to observe that the surface morphology is homogeneous without imperfections or crystallization zones, offering potentially excellent adherence to the substrate. Samples with various D:A weight ratios were evaluated and presented similar surface morphology characteristics. This suggested an excellent intermolecular interaction between the active organic compounds (DCVT-BTT and PC_61_BM), which is relevant to obtain D:A interfaces that allow an efficient photovoltaic process.

**Figure 7:**
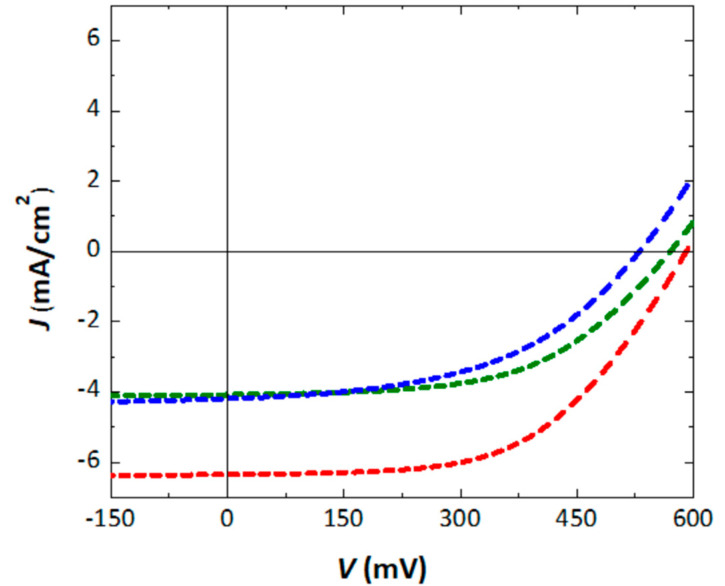
J−V curves obtained from the evaluation of the SM-OSCs with DCVT-BTT:PC_61_BM under AM 1.5G illumination at various weight ratios of D:A: 2:1 (green line), 1:1 (red line), and 1:2 (blue line).

Table 4 and Figure 7 report solar cell parameters and J-V curves for SM-OSCs fabricated using DCVT-BTT:PC_61_BM at various D:A ratios. The device based on DCVT-BTT:PC_61_BM blend film with a D:A weight ratio of 2:1 possesses a PCE of 1.09% with a Voc of 570 mV, a Jsc of 4.07 mA cm^−2^, as well as an FF of 47.15%. For the blend film with a D:A weight ratio of 1:2, the PCE value decreased to 1.03% with a Voc of 530 mV, a Jsc of 4.21 mA cm^−2^, and an FF of 45.96%. Finally, the blend film with a D:A weight ratio of 1:1 showed a PCE of 2.04% (the highest observed in this work) with a Voc of 591 mV, a high Jsc of 6.33 mA cm^−2^, and an FF of 54.64%. The differences observed in the photovoltaic behavior for the OSCs prepared using DCVT-BTT:PC_61_BM at various weight ratios are mainly related to an effect of saturation of active species, either donors or acceptors, which causes an easier recombination of excitons, reducing its photovoltaic properties [71,72,73]. In this sense, it is possible to observe that, as the weight ratio of active components increases, the V_OC_ value decreases. These studies demonstrate that the designed molecule presents an electron donor behavior in heterojunction solar cells, due to its molecular structure, electronic properties, and bulk distribution of their components.

According to the results presented in the previous sections and with respect to the dynamics of electron transfer between donor and acceptor species, it is quite clear that the most efficient factor achieved for the 1:1 ratio is directly related to the increase in the number of electrons that are transferred through the interface, in relation to the strong interaction between DCVT-BTT and PC_61_BM and leading to a decrease in the recombination of charge carriers. Moreover, the relative position of the energy bands of these molecules favors the direct injection of charge carriers.

## 4. Conclusions

In the present study, 2,5,8-Tris[5-(2,2-dicyanovinyl)-2-thienyl]-benzo[1,2-b:3,4-b′:6,5-b″]-trithiophene (DCVT-BTT) was successfully synthesized and characterized. The optical experimental characterization of the compound showed a UV–vis absorption spectrum with an intense absorption band located at a wavelength of ~544 nm that corresponds to the allowed HOMO-LUMO electronic transition in agreement with theoretical calculations. Electronic excitation studies of DCVT-BTT showed favored charge transfer properties when these molecules are stacked, compared to when using monomer molecules only. Regarding the obtained theoretical charge transport properties, the bandgap determination, and the preliminary tests of the electrical behavior of this compound in a bulk heterojunction solar cell, the DCVT-BTT molecule displayed a remarkable behavior as a P-type organic semiconductor. Using phenyl-C61-butyric acid methyl ester (PC_61_BM) as an N-type organic semiconductor, the fabrication of small-molecule organic solar cells (SM-OSC) was carried out for its efficiency evaluation. This type of assembly exhibited a power conversion efficiency (PCE) of 2.04% at a donor: acceptor weight ratio of 1:1. It is important to note that new cell architectures, which consider the stacking of DCVT-BTT molecules, could potentially increase this efficiency.

## Figures and Tables

**Table 1:** Energy values obtained for frontier molecular orbitals (HOMO and LUMO) determined experimentally and theoretically by molecular modeling in acetone as continuum.

Frontier Molecular Orbital	Theoretical Energy with M06-2X/B3LYP Functionals (eV)	Experimental Energy (eV)
HOMO	−7.12/−6.04	−5.06
LUMO	−3.05/−3.61	−3.21

**Table 2:** Electron excitation properties obtained for monomer and dimer of DCVT-BTT.

DCVT-BTT	Trans	Δr(Å)	D(Å)	t(Å)	H(Å)	E_coulomb_ (eV)	S_r_ (a.u.)	Λ(Å)	HDI	EDI	LE(%)	CT(%)
Monomer	S0→S1	1.95	1.68	−2.30	6.25	2.75	0.708	0.698	4.53	4.44	27.6	72.4
Dimer		2.13	2.11	−0.320	6.27	2.29	0.580	0.570	3.56	3.52	38.8	61.2

**Table 3:** Theoretical charge transport parameters obtained from DCVT-BTT dimer using the hopping mechanism. Subscripts h and e correspond to holes and electrons, respectively.

[*t* HOMO] (eV)	λ_h_ (eV)	[*t* LUMO] (eV)	λ_e_ (eV)	*l* (cm)	*k*_ET h_ (seg^−1^)	*k*_ET *e*_ (seg^−1^)	*µ_h_* (cm^2^/V∗s)	*µ_e_* (cm^2^/V∗s)
0.0200	0.4650	0.0030	0.5820	3.32 × 10^8^	1.07 × 10^10^	7.34 × 10^8^	4.60 × 10^−3^	3.16 × 10^−5^

**Table 4:** Circuit voltage (V_OC_), short-circuit current density (J_SC_), and fill factor (FF) solar cell parameters for three different combinations of weight ratio of DCVT-BTT:PC_61_BM active compounds.

Compounds (Weight Ratio)	J_SC_ (mA cm^−2^)	V_OC_ (V)	FF (%)	PCE (%)
DCVT-BTT:PC_61_BM (2:1)	4.07	0.570	47.15	1.09
DCVT-BTT:PC_61_BM (1:1)	6.33	0.591	54.64	**2.04**
DCVT-BTT:PC_61_BM (1:2)	4.21	0.530	45.96	1.03

## Data Availability

Not applicable.

## Supplementary Materials

The following supporting information can be downloaded at: https://www.mdpi.com/article/10.3390/ma16103759/s1, Figure S1: Natural transition orbitals (NTOs) of the first singlet excited-state of DCVT-BTT monomer and its eigenvalue. Figure S2: Hole distribution in DCVT-BTT monomer for the excitation S0→S1. Figure S3: Electron distribution in DCVT-BTT monomer for the excitation S0→S1. Figure S4: The charge difference densities for DCVT-BTT monomer on the electronic state transition from the ground state. Figure S5: Natural transition orbitals (NTOs) of the first singlet excited-state of DCVT-BTT dimer and its eigenvalue. Figure S6: Hole distribution in DCVT-BTT dimer for the excitation S0→S1. Figure S7: Electron distribution in DCVT-BTT dimer for the excitation S0→S1. Figure S8: The charge difference densities for DCVT-BTT dimer on the electronic state transition from the ground state. Figure S9: Fourier transform-infrared (FT-IR, cm^−1^) spectrum of DCVT-BTT. Figure S10: ^1^H-NMR spectrum of DCVT-BTT.
