# Supplementary material for: New Benzotrithiophene-Based Molecules as Organic P-Type Semiconductor for Small-Molecule Organic Solar Cells
Source: Materials (Basel). 2023 May 16;16(10):3759. doi: 10.3390/ma16103759 (PMC10222287; doi:10.3390/ma16103759)
Supplement: Supplementary file 1 [file materials-16-03759-s001.zip › materials-2345099-supplementary.pdf]

materials

Supplementary Materials

New Benzotrithiophene-Based Molecule as Organic P-type Semiconductor for Small-Molecule Organic Solar Cells

1. Graphical representations of electronic excitation monomer for DCVT-BTT

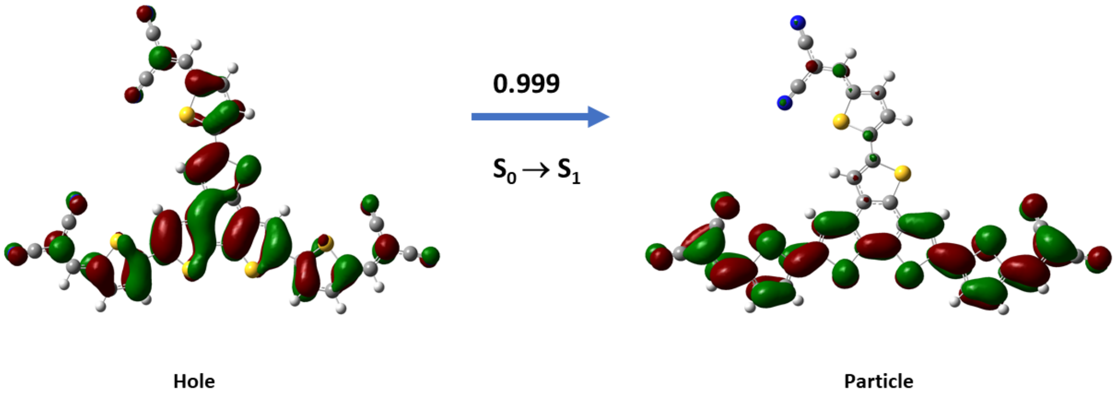

Figure S1. Natural transition orbitals (NTOs) of the first singlet excited-state of DCVT-BTT monomer and its eigenvalue.

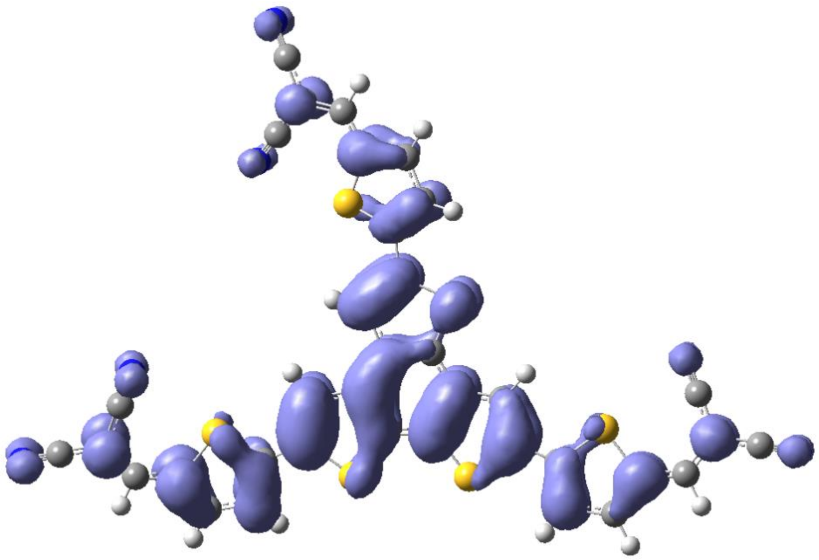

Figure S2. Hole distribution in DCVT-BTT monomer for the excitation $S_0 \rightarrow S_1$.

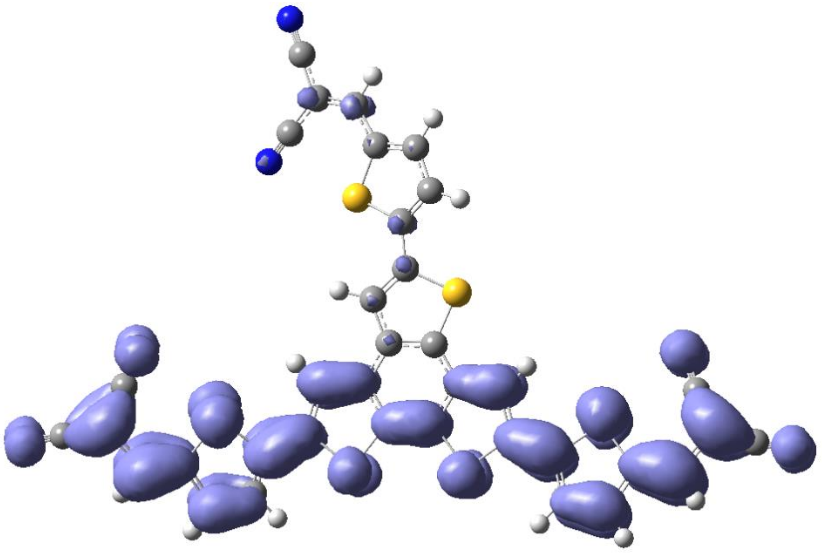

Figure S3. Electron distribution in DCVT-BTT monomer for the excitation $S_0 \rightarrow S_1$.

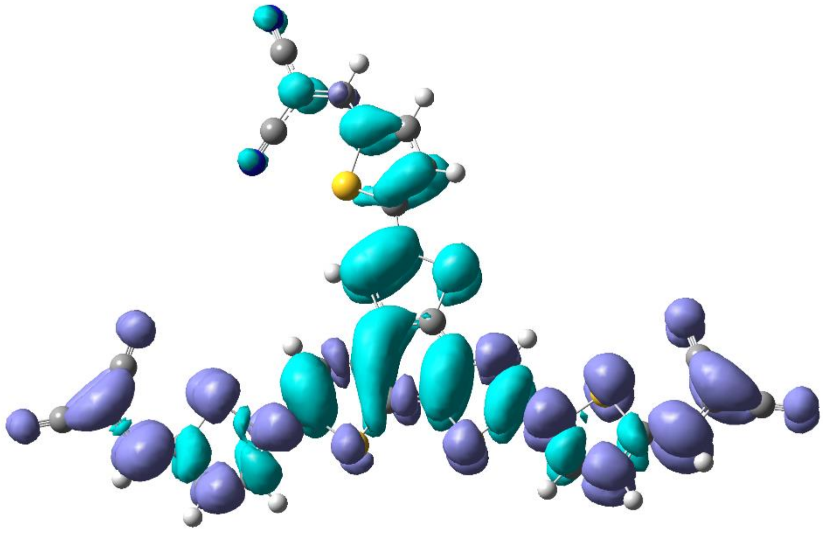

Figure S4. The charge difference densities for DCVT-BTT monomer on the electronic state transition from the ground state.

2. Graphical representations of electronic excitation for DCVT-BTT dimer

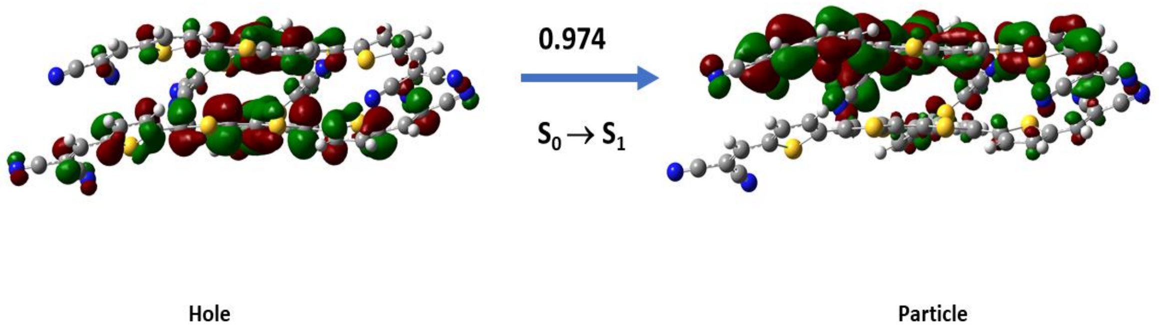

Figure S5. Natural transition orbitals (NTOs) of the first singlet excited-state of DCVT-BTT dimer and its eigenvalue.

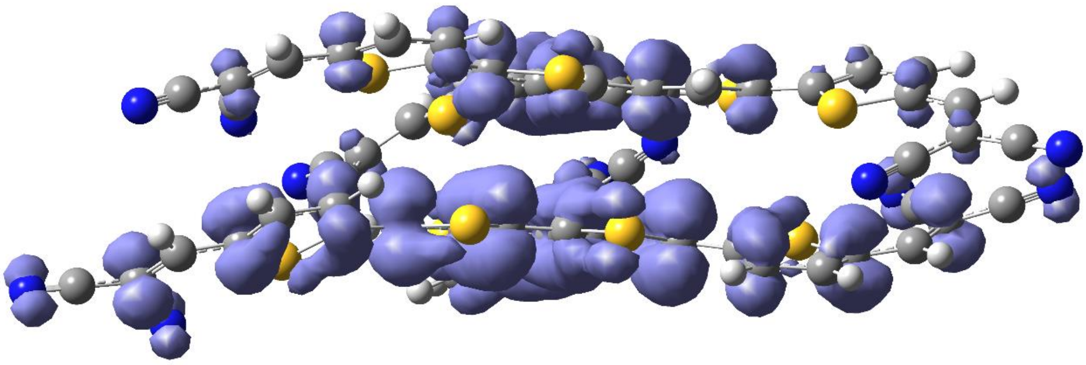

Figure S6. Hole distribution in DCVT-BTT dimer for the excitation $S_0 \rightarrow S_1$.

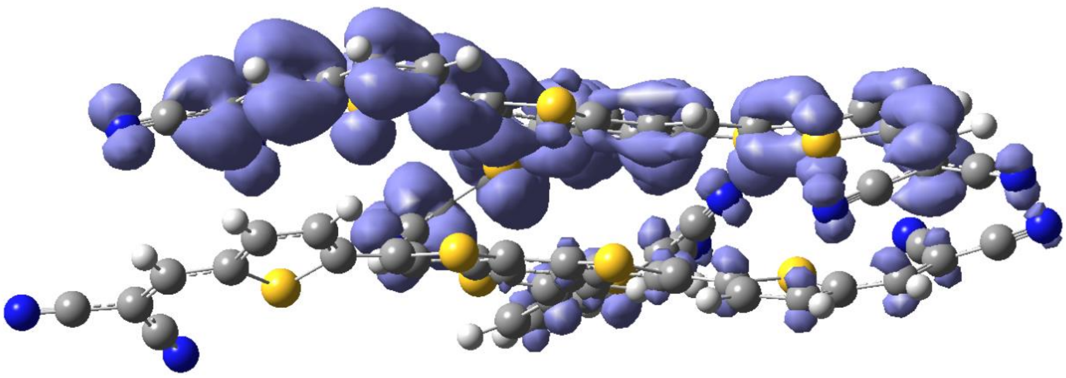

Figure S7. Electron distribution in DCVT-BTT dimer for the excitation $S_0 \rightarrow S_1$.

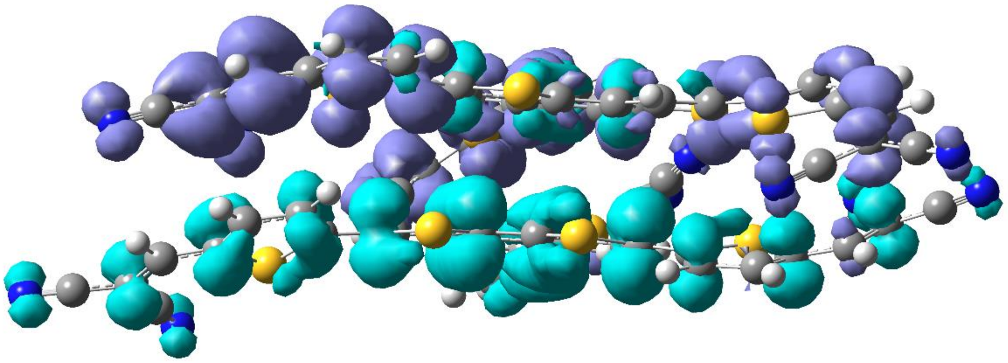

Figure S8. The charge difference densities for DCVT-BTT dimer on the electronic state transition from the ground state.

3. Chemical characterization analysis for DCVT-BTT

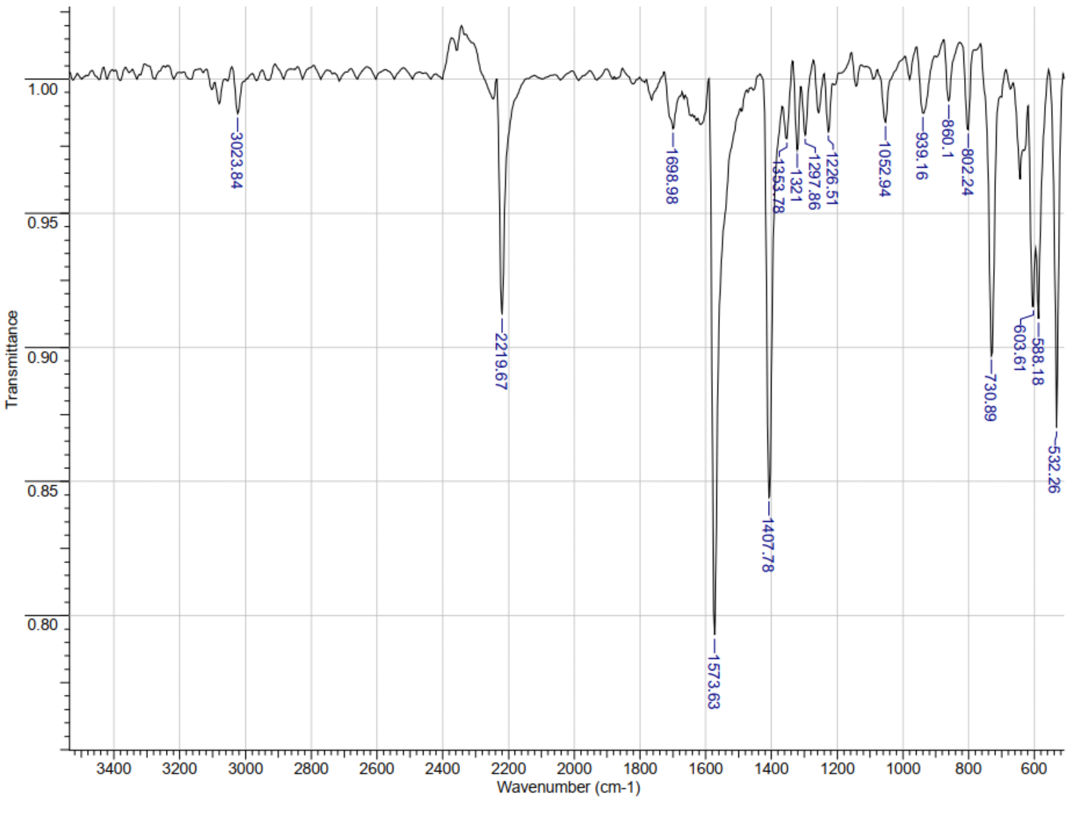

Figure S9. Fourier transform-infrared (FT-IR, cm^{-1}) spectra of **DCVT-BTT.**

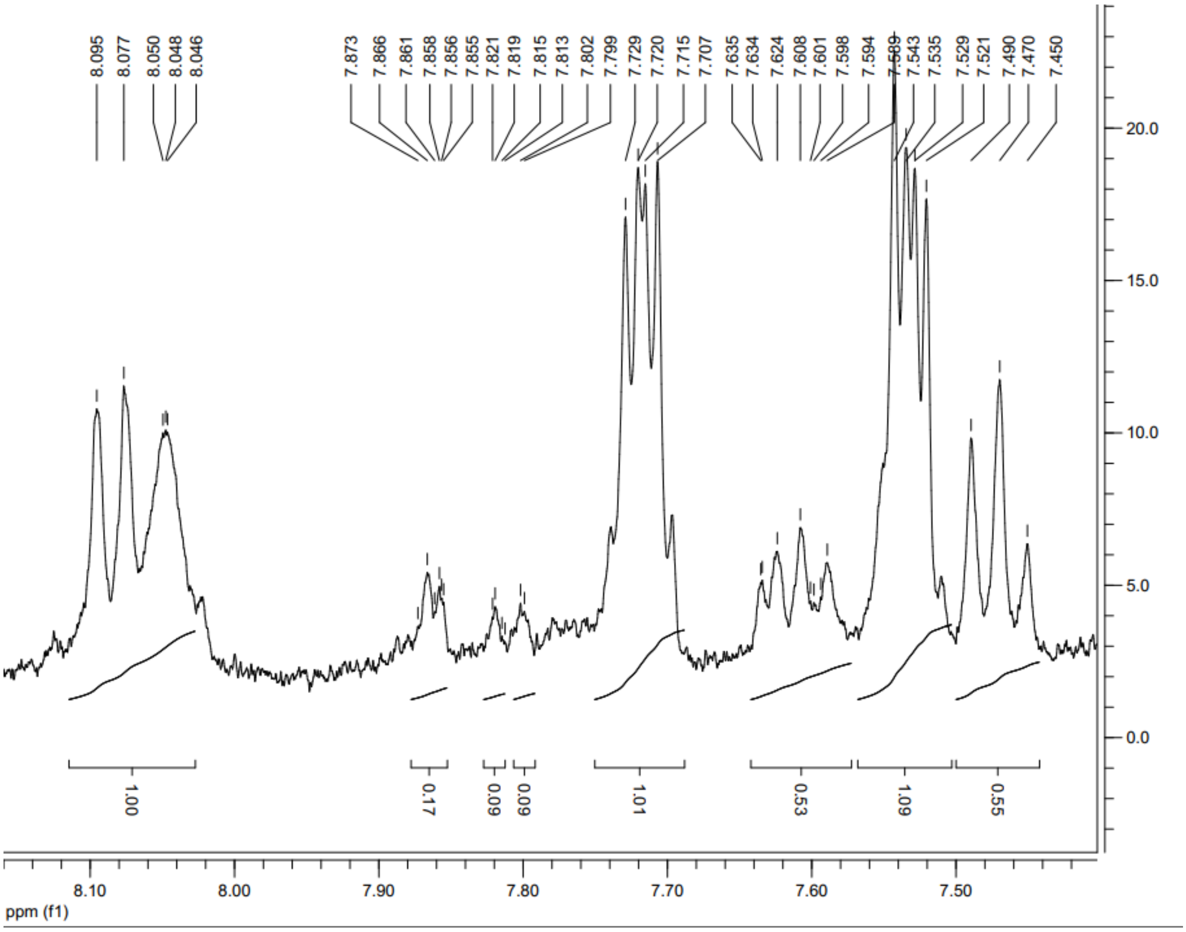

Figure S10. ^{1}H-NMR spectrum of **DCVT-BTT**.
